# Early maladaptive schemas in trichotillomania and skin-picking disorder: their relationships with symptom severity and subtypes

**DOI:** 10.1186/s40359-025-03096-y

**Published:** 2025-07-15

**Authors:** Ella Flagstad, Benjamin Hummelen, Erna Moen, Toril Dammen, Åshild Haaland, Tor Sunde, Bjarne Hansen, Diana Strand Johnsen, Douglas Woods, Torun Grøtte

**Affiliations:** 1 Department of Psychology, NTNU, Trondheim, Norway; 2 Nidaros DPS, St.Olavs University Hospital, Trondheim, Norway; 3 Department of Research and Development, Clinic of Mental Health and Addiction, Oslo University Hospital, Oslo, Norway; 4 Institute of Clinical Medicine, University of Oslo, Oslo, Norway; 5 Department of Psychosocial Health, University of Agder, Kristiansand, Norway; 6 Sørlandet Hospital HF, Kristiansand, Norway; 7 DPS Solvang, Sørlandet Hospital HF, Kristiansand, Norway; 8 Bergen Center for Brain Plasticity, Haukeland University Hospital, Bergen, Norway; 9 Center for Crisis Psychology, Faculty of Psychology, University of Bergen, Bergen, Norway; 10 Department of Psychology, Loyola University Chicago, Chicago, USA

**Keywords:** Trichotillomania, Skin-picking disorder, OCD, Early maladaptive schema, Subtypes

## Abstract

**Background:**

Higher levels of early maladaptive schemas (EMSs) have been associated with increased symptom severity and worse treatment outcomes in several mental disorders, but little is known about the role of EMSs in trichotillomania (TTM) and skin-picking disorder (SPD). The current study therefore aimed to explore the relationship of EMSs with symptom severity and subtypes in patients with TTM and SPD, as well as to compare their baseline EMS levels to those of a group of patients with obsessive‒compulsive disorder (OCD).

**Methods:**

The Young Schema Questionnaire–Short Form (YSQ-SF), along with measures of disorder-specific symptoms and subtypes, was administered to patients with TTM (*n* = 120), SPD (*n* = 75), and OCD (*n* = 88) prior to treatment. Potential between-group differences in EMSs were explored with ANCOVA, with age and illness duration as covariates. Disorder- and subtype-specific patterns were explored with correlational analysis.

**Results:**

No significant baseline differences in EMS severity were found between TTM, SPD, and OCD patients, thereby corroborating EMSs’ transdiagnostic function in mental disorders. Several EMSs were significantly positively associated with self-reported and clinician-rated symptoms and picking/pulling styles of TTM and SPD, with some indications of diagnosis-specific association patterns.

**Conclusion:**

These results suggest that EMSs are common among individuals with TTM and SPD and that assessment of these schemas may enhance clinicians’ understanding of TTM and SPD patients’ presenting problems.

## Introduction

Trichotillomania (TTM) and skin-picking disorder (SPD) are psychiatric disorders characterized by excessive and repetitive hair-pulling or skin-picking. Both disorders are classified as body-focused repetitive behavior disorders (BFRBs) and are included in the obsessive‒compulsive and related disorders chapters (OCRD) of the fifth edition of the Diagnostic and Statistical Manual of Mental Disorders (DSM-5) [1] and the eleventh revision of the International Classification of Diseases (ICD-11) [2]. The diagnostic criteria require that recurrent hair-pulling and skin-picking behavior leads to damage to the body (i.e., significant hair loss or skin lesions) and clinically significant distress or functional impairment [1, 2].

Epidemiological studies estimate that the lifetime prevalence of TTM and SPD ranges from 2 to 4% [3; 4], and psychiatric comorbidities are common. Notably, in a large, representative sample of U.S. adults (*N* = 10,169), 1.7% (50% female) identified as having current TTM [3], with 79% reporting at least one mental health comorbidity, including 24% with comorbid SPD. In individuals with SPD from the same sample (2.1%, 55.4% female), generalized anxiety disorder (63.4%) and depression (53.1%) emerged as the two most common comorbid disorders, and 12.7% had comorbid TTM [4]. TTM and SPD share several clinical characteristics, including symptom presentation (e.g., rituals before and after pulling/picking), mean age of onset (early adolescence), treatment response, and co-occurrence patterns with other mental disorders [5; 6]. Additionally, symptom severity for both hair-pulling and skin-picking tends to peak during the transition to adulthood [7].

Previous literature has identified several subtype characteristics of TTM and SPD [8–10], and one way to understand the various styles of picking/pulling is by differentiating between automatic and focused behavior. Automatic pulling or picking occurs without the individual being aware of it, e.g., while watching TV, reading a book, or driving a car. Focused pulling and picking is usually a more intentional and conscious act occurring as a response to inner experiences, such as a negative emotion, body sensation, or an urge to pull or pick [11]. An ecological momentary assessment study in individuals with SPD (*N* = 57) found that visual or tactile cues and the desire to pick the skin were more important triggers for focused skin-picking, whereas boredom and concentration problems were more prominent in the automatic style [12]. Moreover, emotion regulation difficulties and depression/anxiety symptoms have been more strongly related to the focused style than the automatic style, suggesting that individuals might engage in focused hair-pulling or skin-picking to regulate aversive emotions [13–15]. However, individuals with TTM/SPD often experience both forms of pulling and picking, and they can fluctuate between more focused or more automatic picking/pulling across the lifespan [7, 12].

Research on BFRBs is expanding, but several aspects of these disorders have yet to be fully explored [16], including the relationship between personality traits and the severity of TTM and SPD, as well as variations in pulling and picking styles. Greater insight into personality may enhance our understanding of the clinical manifestations, course of illness, and underlying mechanisms of TTM and SPD, potentially informing the development of more effective treatments [17]. In a study involving 54 individuals with TTM and 25 healthy controls, Keuthen et al. [17] used the NEO Five-Factor Inventory to examine whether normative personality traits could predict TTM diagnosis, hair-pulling severity, perceived control, and focused pulling behavior. The authors found that higher scores of neuroticism significantly predicted all four outcomes, whereas lower scores of agreeableness predicted TTM severity. Interestingly, openness was a positive predictor of TTM severity but a negative predictor of focused pulling. One possible interpretation of this finding is that individuals high in openness may experience stronger urges but may be less likely to act on them due to a greater acceptance of their internal experiences.

While the study by Keuthen et al. [17] offers valuable insights into the role of personality traits in BFRBs, it may be more clinically relevant to focus on personality psychopathology rather than on normative personality traits. Personality psychopathology often has a more direct impact on treatment engagement, symptom persistence, and overall functioning. Several conceptual models of personality psychopathology are linked to specific therapeutic approaches. Broadly speaking, these models can be divided into psychodynamic and cognitive-behavioral traditions. Given that the treatment of BFRBs and other OCRDs typically involves a strong behavioral component, models from the cognitive-behavioral tradition may be particularly relevant.

Among the cognitive-behavioral models, Schema-Focused Therapy has received increasing attention in the treatment of OCRDs [e.g., 18]. Schema-Focused Therapy is grounded in the theory of Early Maladaptive Schemas (EMSs) [19], which can be described as broad themes or patterns consisting of memories, emotions, cognitions, and bodily sensations regarding oneself and one´s relationship with others. EMSs are thought to develop during childhood or adolescence through unmet needs and experiences and negatively influence how individuals experience themselves, others, and the world. Young et al. [19] identified 15 different EMSs that are clustered into five categories called schema domains (see Table 1).

Higher levels of EMSs have been associated with increased symptom severity in several mental disorders, e.g., depression [20], anxiety disorders [21], schizophrenia [22], and OCD [23]. OCD is of particular interest because it has several genetic and phenomenological similarities with TTM and SPD [1, 24, 25]. These similarities were key arguments for classifying TTM and SPD as OCRDs in DSM-5 and ICD-11. However, current knowledge about potential similarities or differences between these disorders in terms of EMSs is sparse. Individuals with OCD have been found to score significantly higher on EMSs compared to both non-clinical and certain clinical control groups (e.g., schizophrenia, panic disorder) [26, 27]. Social isolation, failure, and defectiveness/shame were the most distinguishing EMSs when compared to non-clinical controls, whereas unrelenting standards/ hypercriticalness, approval seeking, negativism, punitiveness, and self-sacrifice were most salient in differentiating OCD from clinical control groups [27]. Although all EMSs have been found to correlate positively with OCD severity, dependence/incompetence, vulnerability to harm or illness, and negativity/pessimism have shown the strongest associations, with correlation coefficients ranging from 0.40 to 0.42 [23]. There is also some evidence for the clinical utility of EMSs in predicting outcome in the treatment of OCD [18, 28]. For instance, Haaland et al. [18] found that higher scores on the abandonment schema at pre-treatment were related to poorer treatment outcome in exposure and response prevention (ERP) for OCD.

To date, only two studies [29, 30] have explored the associations between EMSs and hair-pulling and skin-picking behavior. First, in a cross-sectional study of 59 OCD patients and 26 TTM patients, Lochner et al. [29] explored phenomenological similarities and differences between TTM patients and OCD patients, including EMSs as measured with the Young Schema Questionnaire-Short Form (YSQ-SF) [31]. Compared with TTM patients, OCD patients reported higher levels of five EMSs: mistrust/abuse, social isolation/alienation, defectiveness/shame, subjugation, and emotional inhibition. The authors [29] underlined that these higher scores might be due to the higher rates of comorbidity, disability, and functional impairment in the OCD group. However, the TTM sample size was small (*n* = 26), limiting the generalizability of the findings.

Second, Pozza et al. [30] investigated the relationship between EMSs and skin-picking subtypes in a large community sample of Italian adults (*N* = 596, 66% female). Skin-picking subtypes were measured with the Italian version of the Milwaukee Inventory for Dimensions of Adult Skin Picking (MIDAS). Unlike the original conceptualization of the MIDAS [10], which distinguishes between two subtypes (focused and automatic picking), Pozza et al. [30] also identified a mixed subtype. EMSs were measured with the YSQ-L3, which is a longer version of the YSQ consisting of 232 items and 18 EMSs [19]. Pozza et al. [30] reported that all schemas but one (i.e., emotional deprivation was negatively correlated) correlated positively with all three subtypes (*r* =.12 −.30). Multiple regression analysis revealed that some schemas predicted all subtypes, whereas others were unique predictors for the specific SPD subtypes. For example, higher scores on dependence/incompetence predicted both the focused and automatic subtypes, whereas higher scores on approval/recognition seeking was a specific predictor of the automatic subtype only and higher scores on mistrust/abuse of the focused subtype only. Thus, Pozza et al. [30] concluded that all three skin-picking subtypes were associated with maladaptive cognitive schemas, which might be valuable in the future development of a personalized approach to SPD treatment. These results were somewhat surprising since focused hair-pulling/skin-picking tends to be associated with higher levels of psychopathology, e.g., symptom distress, functional impairment, emotion dysregulation, and pathological attachment style [13, 14, 32, 33]. Further research is therefore needed to determine whether personality problems are indeed similarly distributed across the two picking and pulling styles and whether the findings of Pozza et al. [30] can also be generalized to clinical samples.

In summary, two previous studies investigating the relationship between EMSs and BFRB severity have shown positive associations. However, one study had a small sample size [29], whereas the other involved a non-clinical population [30]. Further research on EMSs is therefore necessary in larger clinical samples of patients with TTM and SPD.

Consequently, the current study aimed to explore the role of EMSs in a large treatment-seeking sample of individuals with TTM (*n* = 120) and SPD (*n* = 75). First, we aimed to compare the EMS scores of the TTM, SPD, and OCD (*n* = 88) groups as measured with the YSQ-SF. Based on the findings of Lochner et al. [29], we hypothesized that OCD patients would score higher on the EMSs mistrust/abuse, social isolation/alienation, defectiveness/shame, subjugation, and emotional inhibition. We had no specific hypothesis regarding the comparison of EMS scores between TTM and SPD patients, as there is currently no theoretical or empirical basis to expect any difference.

Second, we aimed to explore the associations between EMS scores and TTM and SPD severity, as well as between EMS scores and focused and automatic hair-pulling and skin-picking. On the basis of previous studies [e.g., 18; 30], we hypothesized that higher TTM and SPD symptom severity scores would be associated with higher levels of EMSs. Since focused hair-pulling/skin-picking have been associated with higher levels of psychopathology [13–15, 32, 33], we also hypothesized that EMSs would have a stronger association with a focused pulling/picking style.

## Method

### Participants

A total of 283 participants were included in the study: 120 with TTM, 75 with SPD, and 88 with OCD. Patient characteristics are summarized in Table 2. Briefly, the mean age was 30 years in the TTM group, 29 years in the SPD group, and 34 years in the OCD group. The proportion of female participants was 88% in the TTM group, 93% in the SPD group, and 73% in the OCD group.

Inclusion criteria for the TTM and SPD studies were as follows: a diagnosis of TTM or SPD, age between 16 and 67 years, score ≥ 4 on the Clinical Global Impression Scale– Severity (CGI-S) [51], and the ability to understand and write Norwegian. Exclusion criteria were schizophrenia spectrum disorder, developmental disability, or severe sequelae after brain injury. Patients with other mental health conditions such as bipolar disorder, ADHD, autism spectrum disorder, ongoing suicidal ideation, and substance use disorder were included given their symptoms were mild to moderate or acceptably regulated. The severity of comorbid conditions was assessed using structured diagnostic interviews and clinical judgment, with particular attention to whether such conditions could interfere with treatment. In the initial years of the project, suicide risk was assessed by BFRB therapists. From 2021 onward, this responsibility was transferred to the referring clinicians.

The inclusion of OCD participants in the current study was based on the following criteria: a primary diagnosis of OCD; age between 18 and 65 years; score ≥ 16 on the Yale-Brown Obsessive-Compulsive Scale [Y-BOCS; 34]. Participants were excluded if they had an ongoing psychotic episode, if they were abusing/dependent on alcohol or other drugs, if they were using benzodiazepines, if they had a high risk of suicide, or if they had been treated with exposure therapy within the last six months. A total of 48.9% of the OCD sample were using antidepressants at intake, but patients were encouraged to maintain a steady dosage of their medication throughout the treatment period.

### Procedures TTM/SPD sample

Patients with TTM participated in the project “Cognitive Behavioral Group Therapy for TTM,” which began recruitment in 2013 and included its final patient in 2024 (*n* = 160, registered at https://clinicaltrials.gov/, NCT02747771). Similarly, patients with SPD were enrolled in the study “Acceptance-Enhanced Habit Reversal Training in Group Format for Patients with SPD” (not registered at https://clinicaltrials.gov), which started in 2014 and recruited the last patient in 2024 (*n* = 127). Both projects are longitudinal, observational cohort studies with similar procedures, examining TTM and SPD patients undergoing ACT-enhanced habit reversal training within regular clinical care in Norway. Treatment results from a subset of TTM patients from “Cognitive Behavioral Group Therapy for TTM” have already been published [35].

Both projects are approved by the Regional Committee for Medical Research Ethics in Norway (REK-ID: 14368 and 26569) and conducted in accordance with the Declaration of Helsinki [36]. In 2022, the Regional Committee for Medical Research Ethics approved the merging of data of the TTM and SPD project, resulting in “The Norwegian BFRB project”.

Among the 287 patients who were included in The Norwegian BFRB project, 195 completed the YSQ-SF at baseline, constituting the current study sample of TTM and SPD patients. Most of the missing data arose from the exclusion of the YSQ-SF from the questionnaire package during the transition from paper to digital formats in 2022. The present study used cross-sectional, pre-treatment data only. However, the majority of participants were admitted to treatment. Of the 195 patients with SPD or TTM, 17 did not begin treatment, and 15 remained on the waitlist as of August 2023.

Although the primary target group consisted of adults, nine participants aged 16 or 17 were included due to the absence of specialized treatment options for TTM and SPD within Child and Adolescent Psychiatry services in Norway. In accordance with Norwegian regulations, patients who are 16 years or older are permitted to receive treatment within adult psychiatric services. Conversely, patients over the age of 67—the official retirement age in Norway—are typically referred to a geriatric psychiatric outpatient clinic.

From 2014 to 2020, patients could refer themselves to the TTM and SPD projects. During a telephone interview with the leader of the BFRB treatment team, they were screened for both inclusion criteria and exclusion criteria. In addition, a preliminary primary diagnosis of either TTM or SPD was established. From 2021, patients were first referred to a general outpatient clinic, where initial screening took place. Further diagnostic assessment was conducted by a specialist in BFRBs. These clinicians had different professional backgrounds, i.e., clinical psychologist, psychiatrist, or psychiatric nurse, all of whom were trained in BFRB diagnostics by the third (EM) or second last author (DW).

The diagnostic assessments included the Anxiety Disorders Interview Schedule for DSM-IV (used from 2012 to 2017) [37], the Mini International Neuropsychiatric Interview (used from 2018 to 2024) [38], the TTM DSM-IV diagnostic interview [39], and the Keuthen Diagnostic Interview for Skin Picking [40]. Though the presence of comorbid TTM in SPD patients and vice versa was not systematically assessed by a structured diagnostic interview, an additional structured diagnostic interview was administered if there was any indication of comorbid BFRB. As a result, three patients initially referred with a diagnosis of SPD were reclassified as having TTM. In addition, all patients rated the severity of any comorbid hair-pulling or skin-picking problems on a scale from 1 to 10. A score of ≥ 5 was used as an indicator of clinically significant comorbidity. The validity of this self-report measure has not been evaluated.

### Procedures OCD sample

The OCD sample (*n* = 88) is the same sample used in a previous study by Haaland et al. [18], which explored the role of EMSs in predicting the outcome of ERP for OCD. The OCD patients were recruited from one outpatient clinic in Southern Norway where 62 patients completed ERP in a group format, and several outpatient clinics in Middle Norway where 26 participants completed ERP individually. Similar to the TTM and SPD samples, only the pre-treatment scores were used in the present study. Since pre-treatment YSQ-SF scores were available for all OCD patients in the study by Haaland et al. [18], all patients were included also in the current study.

The diagnostic assessment included the Structured Clinical Interview for DSM-IV [41] and the Y-BOCS, which were administered by trained, independent evaluators (i.e., clinical psychologists). The determination of the primary diagnosis was based on the results from the diagnostic interview, self-report questionnaires, and a clinical judgment of the patient’s symptom severity and functional impairment. To assess the level of agreement between raters regarding Axis I diagnoses, audio or videotaped SCID-1 interviews from ten randomly selected patients were reanalyzed by two independent raters recruited and trained at the Department of Psychology, NTNU, in Trondheim. Both raters concurred on the OCD diagnosis for all ten patients. Regarding comorbid diagnoses, discrepancies between the raters were observed in only three cases.

The Regional Ethics Committee for research with human subjects in Norway approved this project. All participants provided written informed consent.

### Measures

The Young Schema Questionnaire–Short Form (YSQ-SF) [31] is a self-report inventory designed to assess EMS. A total of 75 items are used to assess 15 specific EMSs derived from the original longer version of the YSQ [42; 205 items]. Each EMS is represented by five statements that individuals commonly use to describe themselves (see Table 1). These statements are rated on a 6-point Likert scale from 1 (“*completely untrue of me*”) to 6 (“*describes me perfectly*”), where higher scores reflect greater endorsement of the schema domain. The domain scores were calculated as means, and there were no reverse-scored items. The psychometric properties of the YSQ-SF are well established, including the Norwegian version [43, 44].

**Table 1:** Schema domains and early maladaptive schemas in the YSQ-SF [31]

Schema domain	Schema	Description
Disconnection and	Emotional deprivation	The belief that others will not give emotional support.
Rejection	Abandonment/instability	The belief that important others will leave.
	Mistrust/abuse	The belief that one will be exploited by others.
	Social isolation/alienation	The assumption of not belonging to others.
	Defectiveness/shame	The belief of being worthless to others.
Impaired autonomy	Failure	The belief that one is incompetent compared to others.
and performance	Dependence/incompetence	The assumption that one can´t take care of oneself.
	Vulnerability to harm or illness	Expectation that an accident or illness is imminent.
	Enmeshment/undeveloped self	The feeling of fusion identity with important others.
Other-directedness	Subjugation	The feeling that other´s needs are more important.
	Self sacrifice	Attention to other needs at the expense of oneself.
Overvigilance and	Emotional inhibition	The assumption that one must not show emotions.
Inhibition	Unrelenting standards/hypercriticalness	The belief that one should do everything perfect.
Impaired limits	Entitlement/grandiosity	The belief of being superior to others.
	Insufficient self-control/self-dicipline	Lack of self-control and low frustration tolerance.

The Massachusetts General Hospital Hairpulling Scale (MGH-HPS) [45] is a 7-item self-report instrument that measures the severity of hair-pulling in the past week. The first three questions assess the frequency and intensity of the urge to pull hair and the ability to control the urge. The last four questions assess the frequency of and associated distress with hair-pulling, as well as the attempts to resist hair-pulling and how successful these attempts were. Each item is rated on a 5-point Likert scale from 0 to 4, resulting in a total score between 0 and 28. Higher scores indicate increased severity of hair-pulling. The MGH-HPS has shown acceptable psychometric properties, e.g., good internal consistency and construct validity, but slightly low test‒retest reliability [46; 47].

Skin Picking Scale-Revised (SPS-R) [48] is a self-report measure of skin-picking severity over the past week. It consists of eight items, where the first four assess the frequency and intensity of picking urges, the amount of time spent picking, and perceived control over skin-picking. The last four items measure the degree of distress, functional impairment, avoidance, and skin damage related to skin-picking [49]. Each item is rated on a 5-point Likert scale from 0 (“*none*”) to 4 (“*extreme*”). The total score ranges from 0 to 32, with higher scores indicating greater severity of skin-picking. The SPS-R has shown good psychometric properties and is a reliable and valid tool for assessing skin-picking [48]. It has shown acceptable diagnostic accuracy, with a total score of 9 as a suggested cutoff [49].

Milwaukee Inventory for Subtypes of Trichotillomania– Adult Version (MIST-A) [8] is a 15-item self-report instrument that assesses to what degree hair-pulling in TTM is focused or automatic. Each item is rated from 0 (*“not true for any of my pulling”*) to 9 (*“true for all of my hair-pulling”*), with a total range between 0 and 135. The questions are related to the intention and awareness of the pulling and whether the pulling happened as a response to an urge or a negative emotional state. Despite some uncertainty about its factor structure [50], we adopted the original two-factor model proposed by Flessner et al. [8], which includes a 10-item focused pulling subscale and a 5-item automatic pulling subscale. Items from each subscale are summed to form a focused total score (0–90) and an automatic total score (0–45), where higher scores indicate a higher endorsement of focused and automatic hair-pulling, respectively. The MIST-A has shown acceptable reliability and validity [8].

Milwaukee Inventory for Dimensions of Adult Skin Picking (MIDAS) [10] is a 12-item self-report measure designed to assess the degree to which skin-picking is focused or automatic. Each item is rated from 1 (*“not true of any of my skin-picking”*) to 5 (*“true for all of my skin-picking”*). The questions are related to the intention of the picking, whether the picking happened as a response to an urge or a negative emotional state, and the awareness of the picking. Four items measure the degree of automatic skin-picking, whereas eight items measure the degree of focused skin-picking. Items from each subscale are summed to yield a focused score (8–40) and an automatic score (4–20), where higher scores indicate a higher endorsement of focused and automatic skin-picking, respectively. The reliability and validity of this instrument have been demonstrated to be good [10].

Clinical Global Impression Scale– Severity (CGI-S) [51] is an observer-rated scale that assesses the severity of the illness [51] on a scale from 1 (“*normal”*) to 7 (*“among the most severely ill patients”*). It has demonstrated good interrater reliability and validity for several mental disorders [e.g., 52–54], including TTM [35]. In the present study, experienced clinicians assessed each participant with a CGI-S scale modified for TTM and SPD [55]. The OCD patients were not assessed with the CGI-S.

The Yale-Brown Obsessive Compulsive Scale (Y-BOCS) [34] is a 10-item clinician-rated scale designed to assess the severity of obsessive‒compulsive symptoms. Five aspects of both obsessions and compulsions are rated: frequency, interference, distress, resistance, and control. Each item is rated on a 5-point Likert scale ranging from 0 (“*none”*) to 4 (“*extreme”*), with a total range of 0–40. A total score of 8–13 was considered subclinical OCD, 14–21 was considered mild OCD, 22–29 was considered moderate OCD, and 30–40 was considered severe OCD [56]. The psychometrics of the Y-BOCS are well established [e.g., 57, 58].

### Statistical analyses

All the statistical analyses were performed via IBM SPSS Statistics 29. Prior to analysis, the dataset was examined for missing values and skewness. There were no missing YSQ-SF scores in the OCD sample, but 92 participants in “The Norwegian BFRB project” had missing YSQ-SF data. There were no significant differences between the final study sample of TTM and SPD patients (*n* = 195) and the total TTM/SPD sample with missing YSQ-SF scores (*N* = 287) in terms of age (*t* = 0.51, *p* =.609), sex (χ^2^ = 0.65, *p* =.421), or symptom severity as measured by the CGI-S (*t* = -0.75, *p* =.452). Visual inspection and skewness analysis of YSQ-SF, MGH-HPS, SPS-R, MIST-A, and MIDAS indicated moderate skewness for MGH-HPS (-0.61) and YSQ-SF (0.52), whereas the skewness for the remaining variables ranged from − 0.28 − 0.02. These values were considered acceptable.

First, the sociodemographic and clinical characteristics of the three patient groups (i.e., TTM, SPD, and OCD) were compared via the *chi*-square test and one-way analysis of variance (ANOVA). To determine exactly which groups differed from each other, post hoc z tests for independent proportions with Bonferroni corrections were performed after the *chi*-square tests. A post hoc test with Tukey HSD correction was performed after the ANOVA (i.e., comparison of age).

Second, several one-way analyses of covariance (ANCOVAs) with Bonferroni correction were carried out to compare the total YSQ score and the score of the 15 EMSs among patients with TTM, SPD, and OCD. Owing to 16 comparisons, the critical alpha level was set at 0.003. Age and illness duration were included as covariates. Due to missing data on illness duration, eleven participants (i.e., 4 TTM patients, 6 SPD patients, 1 OCD patient) were excluded from the ANCOVAs. Levene’s tests for all EMS variables were non-significant, indicating that the assumption of homogeneity of variances was met. The assumption of homogeneity of regressions slopes was also met, as no significant interactions were found between group membership and the two covariates.

Finally, to examine the relationships between the symptom severity of TTM and SPD and EMSs, Pearson´s bivariate correlation coefficients were calculated between the MGH-HPS, SPS-R, CGI-S, and YSQ-SF scores. Similarly, correlational analysis between the MIST-A, MIDAS, and YSQ-SF scores was performed to explore the relationships between EMSs and the two picking/pulling styles of TTM and SPD: automatic versus focused. The correlation coefficients were considered small at 0.10, moderate at 0.30, and strong at 0.50 [59].

## Results

### Participant characteristics

The demographic and clinical characteristics of the TTM, SPD, and OCD patients are presented in Table 2. Significant group differences were found in terms of age, sex, employment/student status, marriage/cohabitant status, and psychiatric comorbidities. The TTM and SPD patients were younger than the OCD patients were. Women outnumbered men in all three patient groups, but the predominance of women was significantly greater in the TTM and SPD groups than in the OCD group. All three groups had high rates of psychiatric comorbidity (ranging from 62.5 to 79.4%), but the TTM group demonstrated significantly higher rates of any comorbid psychiatric disorder, particularly depressive disorders, than did the OCD group. Employment status was significantly greater in the TTM and SPD groups than in the OCD group (medium effect size).

In terms of psychiatric comorbidity within the three groups, 12.4% of the TTM sample and 27.0% of the SPD sample had comorbid OCD, *x*^*2*^(1, *n* = 160) = 5.50, *p* =.019, *d* = 0.19. The presence of BFRBs was not diagnosed in the OCD sample, so the proportion of OCD patients with comorbid TTM or SPD is unknown. The proportion of self-identified comorbid skin-picking problems in TTM patients was 19.8%, whereas the proportion of self-identified hair-pulling problems in SPD patients was 16.4%.

The three patient groups’ mean duration of illness ranged from 15.4 to 17.7 years. The OCD patients had a mean Y-BOCS score of 24.3 (*SD* = 4.2), which corresponds to moderate to severe obsessive‒compulsive symptom severity. The TTM patients had a mean MGH-HPS score of 17.9 (*SD* = 4.6), whereas the SPD patients had a mean SPS-R score of 17.3 (*SD* = 3.7). TTM symptom severity classification on the basis of the MGH-HPS does not exist yet, but the mean SPS-R total score was above the suggested clinical cutoff of nine. The CGI-S scores of the TTM and SPD patients did not significantly differ from each other, with a mean severity score corresponding to “markedly ill”.

### Comparison of EMS severity: TTM vs. SPD vs. OCD

The TTM, SPD, and OCD patients’ mean pre-treatment scores of YSQ-SF total score and 15 EMSs are presented in Table 3, adjusted for age and duration of illness. A comparison of EMSs among the three patient groups revealed no significant group differences in any of the 15 schemas.

**Table 2:** Comparison of sociodemographic and clinical participant characteristics: TTM vs. SPD vs. OCD

Characteristics	TTM	SPD	OCD				
	(*n* = 120)	(*n* = 75)	(*n* = 88)				
	%	%	%	*x* ^*2*^	*p*	*Phi*	Post hoc
Female	88.3	93.3	72.7	15.36	< 0.001	0.23	TTM, SPD > OCD
Employed/student	88.2^a^	85.3	58.0	30.24	<0.001	0.33	TTM, SPD > OCD
Married/cohabitant	47.5	37.8^b^	64.8	12.35	0.002	0.21	OCD > TTM, SPD
Any comorbid psychiatric disorder^j^	79.4^c^	69.8^d^	62.5	6.43	0.040	0.16	TTM > OCD
Any depressive disorder	56.7	39.7	34.1	10.30	0.006	0.20	TTM > OCD
Any anxiety disorder	60.8	50.8	48.9	3.01	0.222	0.11	
	*M (SD)*	*M (SD)*	*M (SD)*	*F* or *t*	*p*	*η2* or *d*	
Age	30.2 (10.1)	29.3 (7.7)	34.4 (11.5)	6.33	0.002	0.04	OCD > TTM, SPD
Illness duration	17.7 (11.3)^e^	15.9 (8.6)^f^	15.4 (11.1)^g^	1.32	0.268	0.01	
CGI-S	5.0 (0.7)^h^	4.8 (0.7)^i^		1.71	0.089	0.27	

Note. TTM = trichotillomania; SPD = skin-picking disorder; OCD = obsessive‒compulsive disorder; MGH‒HPS = Massachusetts General Hospital Hairpulling Scale;

SPS‒R = Skin Picking Scale Revised; Y‒BOCS = Yale‒Brown Obsessive‒Compulsive Scale; CGI-S = Clinical Global Impression Scale - Severity. ^a^ *n* = 119, ^b^ *n* = 74, ^c^ *n* = 97, ^d^ *n* = 63, ^e^ *n* = 116, ^f^ *n* = 69, ^g^ *n* = 87, ^h^ *n* = 105, ^i^ *n* = 69, ^j^ To match the calculation of psychiatric comorbidity in the OCD sample in Haaland et al. [18], this category includes depressive disorders, anxiety disorders, PTSD, and eating disorders

**Table 3:** ANCOVA: comparison of EMSs among individuals with TTM, SPD, and OCD, controlled for age and illness duration

Schema	TTM (*n* = 116)	SPD (*n* = 69)	OCD (*n* = 87)			
	*M (SE)*	*M (SE)*	*M (SE)*	*F* ^*a*^	*p*	*ηp* ^*2*^
YSQ-SF total score	35.7 (1.0)	37.5 (1.4)	34.7 (1.3)	1.06	0.347	0.008
Emotional deprivation	2.1 (0.1)	2.3 (0.1)	1.9 (0.1)	2.02	0.134	0.015
Abandonment/instability	2.5 (0.1)	2.8 (0.2)	2.6 (0.2)	0.60	0.552	0.004
Mistrust/abuse	2.1 (0.1)	2.3 (0.1)	1.9 (0.1)	2.40	0.093	0.018
Social isolation/alienation	2.5 (0.1)	2.8 (0.2)	2.2 (0.2)	2.96	0.054	0.022
Defectiveness/shame	2.2 (0.1)	2.3 (0.2)	2.0 (0.2)	0.75	0.474	0.006
Failure	2.3 (0.1)	2.6 (0.2)	2.1 (0.1)	1.97	0.142	0.015
Dependence/incompetence	1.8 (0.1)	1.9 (0.1)	2.1 (0.1)	1.30	0.276	0.010
Vulnerability to harm or illness	2.1 (0.1)	2.4 (0.1)	2.4 (0.1)	1.85	0.159	0.014
Enmeshment/undeveloped self	1.8 (0.1)	1.8 (0.1)	1.9 (0.1)	0.11	0.804	0.002
Subjugation	2.3 (0.1)	2.5 (0.1)	2.5 (0.1)	0.73	0.483	0.005
Self sacrifice	3.3 (0.1)	3.0 (0.1)	3.2 (0.1)	1.51	0.223	0.011
Emotional inhibition	2.3 (0.1)	2.5 (0.1)	2.1 (0.1)	1.48	0.229	0.011
Unrelenting standards/hypercriticalness	3.4 (0.1)	3.6 (0.1)	3.3 (0.1)	0.50	0.607	0.004
Entitlement/grandiosity	2.1 (0.1)	2.0 (0.1)	2.0 (0.1)	0.25	0.776	0.002
Insufficient self-control/self-dicipline	2.7 (0.1)	2.9 (0.1)	2.3 (0.1)	4.91	0.008	0.035

Note. TTM = trichotillomania; SPD = skin-picking disorder; OCD = obsessive‒compulsive disorder

^a^*df* = 2, 267

To explore how comorbidity between TTM, SPD, and OCD might have influenced the results, the ANCOVAs were rerun after removing 59 patients who had comorbidities from the other groups, i.e., 23 TTM patients with a self-identified skin-picking problem, 12 SPD patients with a self-identified hair-pulling problem, and 29 TTM and SPD patients with comorbid OCD^1^. The results of the ANCOVAs can be found in Table 4. No significant differences in EMS scores were found between the “pure” cases of TTM, SPD, and OCD either.

**Table 4:** ANCOVA: comparison of EMSs among individuals with TTM, SPD, and OCD without comorbidities, controlled for age and illness duration

Schema	TTM (*n* = 88)	SPD (*n* = 48)	OCD (*n* = 87)			
	M (SE)	M (SE)	M (SE)	F^a^	*p*	ɳp^2^
YSQ-SF total score	34.1 (1.3)	35.4 (1.8)	34.7 (1.3)	0.19	0.826	0.002
Emotional deprivation	2.1 (0.1)	2.3 (0.2)	1.9 (0.1)	1.38	0.254	0.013
Abandonment/instability	2.3 (0.1)	2.5 (0.2)	2.7 (0.1)	1.14	0.322	0.011
Mistrust/abuse	2.0 (0.1)	2.1 (0.2)	1.9 (0.1)	0.59	0.554	0.006
Social isolation/alienation	2.3 (0.1)	2.8 (0.2)	2.2 (0.1)	3.02	0.051	0.028
Defectiveness/shame	2.1 (0.1)	2.1 (0.2)	2.0 (0.1)	0.11	0.896	0.001
Failure	2.1 (0.1)	2.4 (0.2)	2.1 (0.1)	1.00	0.370	0.009
Dependence/incompetence	1.7 (0.1)	1.7 (0.2)	2.1 (0.1)	2.80	0.063	0.026
Vulnerability to harm or illness	2.0 (0.1)	2.0 (0.2)	2.4 (0.1)	2.40	0.093	0.022
Enmeshment/undeveloped self	1.7 (0.1)	1.6 (0.2)	1.9 (0.1)	1.51	0.224	0.014
Subjugation	2.2 (0.1)	2.3 (0.2)	2.5 (0.1)	1.02	0.363	0.010
Self sacrifice	3.2 (0.1)	3.0 (0.2)	3.2 (0.1)	0.98	0.378	0.009
Emotional inhibition	2.2 (0.1)	2.5 (0.2)	2.2 (0.1)	1.13	0.325	0.011
Unrelenting standards/hypercriticalness	3.3 (0.1)	3.5 (0.2)	3.3 (0.1)	0.63	0.535	0.006
Entitlement/grandiosity	2.1 (0.1)	1.9 (0.1)	2.0 (0.1)	0.47	0.629	0.004
Insufficient self-control/self-dicipline	2.7 (0.1)	2.7 (0.2)	2.3 (0.1)	2.46	0.088	0.023

Note. YSQ-SF = Youngs Schema Questionnaire Short Form; TTM = trichotillomania; SPD = skin-picking disorder; OCD = obsessive‒compulsive disorder. ^a^*df* = 2, 210

### Relationships between EMSs and TTM and SPD severity

The pre-treatment correlations between EMSs and the severity of TTM and SPD are presented in Table 5. The YSQ-SF total score correlated significantly positively with self-reported TTM and SPD severity (MGH-HPS and SPS-R) and clinician-based (CGI-S) measure of TTM severity. These correlations were of small to moderate strength (*r* =.26 −.32). In the TTM sample, 10 out of 15 EMSs were significantly positively correlated with higher MGH-HPS scores, and seven EMSs were significantly positively correlated with higher CGI-S-TTM scores. The correlation coefficients ranged from 0.20 to 0.34, indicating associations of small to moderate strength.

In the SPD sample, 5 out of 15 EMSs were significantly positively correlated with the SPS-R, and one EMS was significantly positively correlated with the CGI-S-SPD. The correlation coefficients were in the same range as the TTM measures, i.e., 0.23 − 0.33.

Exploring the pattern of correlation coefficients, four of the EMSs were significantly positively associated with both TTM and SPD severity, with failure and self-sacrifice as the two strongest associations. Emotional deprivation, mistrust/abuse, social isolation/alienation, subjugation, and emotional inhibition were associated solely with TTM severity, whereas abandonment/instability and unrelenting standards/hypercriticalness were correlated with SPD severity alone.

**Table 5:** Pearson’s correlations between EMSs and TTM and SPD severity

Schema	MGH-HPS	CGI-S - TTM	SPS-*R*	CGI-S - SPD
	(*n* = 118)	(*n* = 105)	(*n* = 73)	(*n* = 69)
YSQ-SF total score	0.32**	0.27*	0.26*	0.20
Emotional deprivation	0.21*	0.12	0.09	0.09
Abandonment/instability	0.18	0.17	0.25*	0.20
Mistrust/abuse	0.26**	0.23*	0.17	0.19
Social isolation/alienation	0.29**	0.27**	0.08	0.11
Defectiveness/shame	0.23*	0.24*	0.23*	0.19
Failure	0.34**	0.32**	0.27*	0.02
Dependence/incompetence	0.16	0.18	0.15	0.08
Vulnerability to harm or illness	0.22*	0.10	0.24*	0.15
Enmeshment/undeveloped self	0.26**	0.05	0.12	0.25*
Subjugation	0.32**	0.21*	0.15	0.01
Self sacrifice	0.24*	0.20*	0.33**	− 0.04
Emotional inhibition	0.25**	0.17	− 0.11	0.07
Unrelenting standards/hypercriticalness	0.00	0.24*	0.21	0.21
Entitlement/grandiosity	0.05	− 0.07	0.10	0.10
Insufficient self-control/self-dicipline	0.11	0.16	0.10	0.12

Note. YSQ-SF = Youngs Schema Questionnaire Short Form; MGH-HPS = Massachusetts General Hospital Hairpulling Scale; SPS-R = Skin Picking Scale Revised; CGI-S = Clinical Global Impression Scale - Severity *p <.05, **p <.01

### Relationships between EMSs and focused and automatic hair-pulling/skin-picking

Pre-treatment correlations between EMSs and focused and automatic hair-pulling and skin-picking styles in the TTM and SPD groups are presented in Table 6. In the TTM sample, the YSQ-SF total score was significantly positively correlated with the focused hair-pulling style only (i.e., MIST-A focused). This relationship was of moderate strength (*r* = 0.32). Further investigation of the specific EMSs revealed that 9 out of 15 EMSs were significantly positively correlated with a focused hair-pulling style, with a range in *r* from 0.21 to 0.35, thereby indicating associations of small to moderate strength. The EMSs with the strongest associations with focused hair-pulling were abandonment/instability, failure, and social isolation/alienation. The automatic hair-pulling style had only one significant association with EMSs, i.e., a small, positive correlation with enmeshment/undeveloped self (*r* = 0.25).

In the SPD sample, the YSQ-SF total score and 3 out of 15 specific EMSs were significantly positively correlated with the focused skin-picking style (i.e., MIDAS focused): abandonment/instability, mistrust/abuse and defectiveness/shame. These correlations ranged from 0.24 to 0.32, thereby indicating associations of small to moderate strength. The automatic skin-picking style was not significantly correlated to any of the EMSs.

**Table 6:** Pearson’s correlations between EMSs and focused and automatic picking/pulling styles in TTM and SPD

Schema	Focused hairpulling	Automatic hairpulling	Focused skin-picking	Automatic skin-picking
	(*n* = 111^a^)	(*n* = 113^a^)	(*n* = 74)	(*n* = 74)
YSQ-SF total score	0.32**	0.13	0.24*	0.16
Emotional deprivation	0.04	0.13	− 0.00	0.17
Abandonment/instability	0.35**	0.06	0.30*	0.13
Mistrust/abuse	0.26**	0.15	0.24*	0.15
Social isolation/alienation	0.32**	0.15	0.07	0.15
Defectiveness/shame	0.24*	0.10	0.32**	0.23
Failure	0.33**	− 0.00	0.17	0.10
Dependence/incompetence	0.26**	0.09	0.15	0.08
Vulnerability to harm or illness	0.24**	0.07	0.17	0.12
Enmeshment/undeveloped self	0.21*	0.25*	0.17	0.07
Subjugation	0.24**	0.08	0.11	0.06
Self sacrifice	0.13	0.06	0.15	− 0.00
Emotional inhibition	0.17	0.13	− 0.04	0.05
Unrelenting standards/hypercriticalness	0.12	0.11	0.12	0.09
Entitlement/grandiosity	0.03	0.07	0.13	0.01
Insufficient self-control/self-dicipline	0.17	− 0.12	0.09	0.00

Note. YSQ-SF = Youngs Schema Questionnaire Short Form. Focused and automatic hairpulling was measured by the Milwaukee Inventory for Subtypes of Trichotillomania– Adult Version. Focused and automatic skin-picking was measured by the Milwaukee Inventory for Dimensions of Adult Skin Picking. ^a^Numbers differ due to missing values in single items *p <.05, **p <.01

## Discussion

This study aimed to investigate the role of EMSs in three OCD-related disorders within a large clinical sample, with a primary focus on TTM and SPD. All diagnostic groups presented elevated levels of EMSs, but there were no significant differences among the OCD, TTM, and SPD groups. Several EMSs were significantly positively associated with self-reported and clinician-rated symptoms and picking/pulling styles of TTM and SPD, with some indications of diagnosis- and subtype-specific association patterns. These results suggest that EMSs are common among individuals with TTM and SPD and that the assessment of EMSs may be of relevance and help to clinicians’ understanding of TTM and SPD patients’ presenting problems [26].

### Comparison of EMSs among the TTM, SPD, and OCD groups

Previous research has identified several significant differences in EMSs between individuals with OCD and those with other psychiatric disorders, such as bipolar disorder, schizophrenia, and panic disorder [27]. Among these, the EMSs of social isolation, abandonment, and vulnerability to harm were particularly salient in distinguishing OCD from clinical control groups. In the present study, however, no significant differences in EMSs were found between patients with OCD, TTM, and SPD, while controlling for age and illness duration. Furthermore, the three patient groups shared a common pattern in which unrelenting standards/hypercriticalness and self-sacrifice were the most highly endorsed EMSs. This finding aligns with previous OCD research, where these two schemas have consistently ranked among the top five most strongly endorsed EMSs in individuals with OCD [27]. In this regard, EMSs align with other phenomenological and genetic similarities among the three disorders, which formed the basis for their inclusion in the OCRD chapter in both the DSM-5 and ICD-11 [24, 25].

Our hypothesis - based on the findings of Lochner et al. [29] - that patients with OCD would score significantly higher than TTM patients on mistrust/abuse, social isolation, defectiveness/shame, subjugation, and emotional inhibition was not supported. However, most of these EMSs were significantly correlated with focused hair-pulling in our study, corroborating our expectations that focused hair-pulling would be more strongly associated with personality problems than automatic hair-pulling [13, 17, 32]. Nevertheless, this does not explain why these EMSs were not elevated in the OCD sample. Differences in sample characteristics could be a possible explanation for this discrepancy; i.e., it might be that the TTM patients in the study of Lochner et al. [29] had higher levels of automatic hair-pulling. Moreover, the small sample size in Lochner et al.’s study [29; *n* = 26] made it highly vulnerable to sampling bias, increasing the likelihood of Type 1 and Type 2 errors, e.g., finding a statistically significant result when there is no true effect.

The lack of between-group differences in EMSs corroborates previous findings that EMSs are prevalent in individuals with a broad range of mental disorders, such as mood and anxiety disorders [60] and psychosis [61]. Moreover, as pinpointed by recent reviews of mental disorder-specific EMS associations [26, 27], it might also indicate the transdiagnostic relevance of EMSs. According to Bär et al. [27], EMSs appear to function *“as transdiagnostic concepts indexing a general vulnerability to psychopathology*,* while specific EMSs (constellations) may index a vulnerability to specific forms of psychopathology”* (p. 741).

### Diagnosis- and subtype-specific association patterns

The pattern of associations between BFRB symptoms and the specific EMSs seemed to be somewhat different in the context of TTM and SPD, both in terms of the number and type of significant associations. Self-reported and clinician-rated TTM severity (i.e., MGH-HPS and CGI-S) correlated significantly positively with 10 and seven of the 15 EMSs, respectively, with failure, subjugation, and social isolation/alienation (*r* = 0.29 − 0.34) as the three schemas showing the strongest associations with TTM symptoms. This suggests that individuals with TTM believe that they are incompetent compared with others, that they do not belong to others, and that others’ needs are more important than their own needs are [19]. The association with social isolation and failure aligns well with the results of previous studies showing that low self-esteem, shame, and avoidance of activities and social situations (including work) are common in TTM [55, 62].

We found fewer significant associations between EMSs and SPD severity, as well as between EMSs and focused skin-picking, than anticipated. Nevertheless, both abandonment/instability and defectiveness/shame schemas were associated with focused skin-picking, suggesting that skin-picking may be a coping strategy for dealing with negative feelings or thoughts, such as worries about abandonment. The same tendency was observed for TTM, where focused pulling had the strongest correlation with abandonment/instability.

Our findings of smaller associations with focused skin-picking contrast with those of Pozza et al. [30], where both the automatic and focused skin-picking subtypes were significantly correlated with all EMSs. This discrepancy may be partly attributable to differences in sample characteristics. In our study, all participants were referred for severe SPD symptoms, whereas Pozza et al.’s [30] study involved a community sample. In clinical samples, individuals often display restricted ranges in psychopathology scores due to heightened symptom severity, which leads to lower variance. This restriction in range can attenuate correlation coefficients, making associations appear weaker than in more heterogeneous samples [63]. Additionally, Pozza et al. [30] used a longer version of the YSQ than we did, and previous studies have shown that different YSQ versions can influence its psychometric properties and results [26, 64].

The differential results between TTM and SPD might be an indication of disorder-specific EMS profiles, with more significant associations between EMSs and TTM symptoms than between EMSs and SPD symptoms. However, the results must be interpreted with caution, as the SPD sample was considerably smaller than the TTM sample, making the results related to individuals with SPD less robust. Moreover, a recent meta-analysis [26] examining the relationships between EMSs and specific mental disorders (*k* = 27; TTM and SPD not included) revealed significant heterogeneity in the results across studies of the same mental disorder. These findings suggest that different mental disorders may not be distinctly characterized by specific EMSs. As such, further studies of the relationship between EMSs and BFRB symptoms are needed to identify whether there is a disorder-specific relationship between EMSs, TTM and SPD and whether EMSs might be related to TTM and SPD treatment outcomes.

### Sociodemographic and clinical participant characteristics

One of the most notable findings in our comparative analyses was the lower level of occupational and educational functioning within the OCD group than in the TTM and SPD groups, which is consistent with previous studies indicating greater lifetime impairment among individuals with OCD (e.g., 29, 65]. In contrast, levels of mental distress were comparable across all groups, as indicated by similar levels of EMSs and similar comorbidity patterns. The TTM group exhibited even slightly greater distress, as reflected by a greater prevalence of depressive disorders. This finding contrasts with the fact that treatment availability for OCD patients is much greater than that for TTM patients and SPD patients. Currently, in Norway, over 30 specialized teams have been established to provide evidence-based treatment for OCD, whereas only one public hospital provides specialized treatment for SPD, and two provide treatment for TTM. A recent study by Capel et al. [66] also revealed limited knowledge of diagnostic criteria and evidence-based treatment for TTM and SPD among mental health providers in the U.S., highlighting the need to increase provider knowledge and competency in treating TTM and SPD. Patients with SPD and TTM deserve equal access to mental health care, comparable to those with OCD.

### Clinical implications

Our findings indicate that both focused hair-pulling and focused skin-picking are associated with elevated levels of abandonment, defectiveness, and shame. While these results do not directly replicate previous studies on the association between EMSs and BFRBs, they are consistent with the findings of Rehm et al. [67], who identified negative self-beliefs as the most prominent factor in individuals with TTM. Together, these findings underscore the importance of addressing negative self-concepts in patients with predominantly focused pulling or picking behavior.

Although Schema-Focused Therapy may be a suitable intervention for these patients, alternative approaches should also be considered. Contemporary models of personality pathology, such as the DSM-5 Alternative Model for Personality Disorders (AMPD) and the ICD-11 model for personality disorder, emphasize impairments in self-functioning and interpersonal functioning as core features of personality disorders. Accordingly, when implementing personality-focused treatment for OCRDs, it may be beneficial to consider therapeutic approaches grounded in the Level of Personality Functioning Scale presented in the DSM-5.

Hutsebaut and Bender [68] recently outlined how the LPFS framework can be applied in everyday clinical practice. For example, difficulties with self-esteem may be addressed by exploring maladaptive self-beliefs, identifying triggers for fluctuations in self-worth, and encouraging reflection on personal needs, values, and perspectives to promote a clearer and more coherent sense of identity. These interventions can be flexibly adapted to match the severity of each patient’s personality dysfunction. For instance, pervasive issues related to abandonment are typically classified as a level 3 impairment in personality functioning, indicating severe impairment of personality functioning. From a practical standpoint, treatment of personality problems within the framework of the AMPD may be more readily implemented in routine clinical settings than more specialized approaches, which often require additional training and theoretical familiarity.

However, in the context of BFRBs, an important modification may be warranted: low self-esteem may not only maintain the behavior but also result from it. Moreover, the relationship between BFRBs and the interpersonal domain of the Level of Personality Functioning Scale remains insufficiently understood. If pulling and picking behaviors are primary drivers of low self-esteem—rather than symptoms of interpersonal dysfunction—then individuals with these behaviors may exhibit relatively fewer difficulties in interpersonal functioning, such as challenges with mentalization or sustaining close relationships. This distinction warrants further empirical investigation.

### Strengths and limitations

This study has several strengths, e.g., it includes a large, clinical sample of individuals with TTM, SPD, and OCD. This study is also the first to examine the associations between EMSs and symptom severity, as well as the the picking and pulling styles in TTM and SPD, and it is based on validated self-reports and clinician-based assessments. It is, however, essential to interpret the results within the context of several study limitations.

First, this study correlates two sets of self-report scales that involve reporting negative aspects about oneself (i.e., hair-pulling and skin-picking behavior and EMSs), which means that the correlation estimates might be overrated owing to shared method variance and self-reporting biases [69]. Moreover, CGI-S was rated by therapists rather than independent raters. Since a CGI-S score of at least 4 was required for study inclusion, therapists evaluating patients for eligibility may have been more inclined to assign a score of 4, even when a score of 3 or lower would have been more appropriate. This potential “inverse leniency bias” [70] could have resulted in reduced standard deviations for the CGI-S and attenuation of the correlations between this measure and other assessments.

Second, all study groups had a predominance of women, and all participants were treatment-seekers. As such, the results may not apply to all individuals with TTM, SPD, and OCD. Third, the SPD sample was smaller than the TTM sample, making the results related to individuals with SPD less robust. Fourth, the comorbidity between SPD and TTM was assessed through self-report measures rather than clinician-administered evaluations, and the presence of TTM and SPD was not diagnosed within the OCD sample. Additionally, comorbid depression and anxiety may have acted as potential confounding variables. However, due to a substantial amount of missing data for these variables, we opted not to include them as covariates in the ANCOVA. Consequently, the lack of significant differences in EMSs across the three diagnostic groups may have been more influenced by psychiatric comorbidities than is currently understood.

Fifth, the cross-sectional design of the current study precludes any conclusions about causal relationships or changes over time. Experimental and longitudinal studies are needed to explore the direction of causation between BFRB symptoms and EMSs.

Finally, it should be noted that the differentiation between focused and automatic pulling/picking styles has been criticized, as has the reliability of the subtype inventories MIST-A and MIDAS [9; 50]. Further research should therefore examine the psychometric properties of the tools used to measure the picking/pulling styles of the TTM and SPD and continue to explore alternative perspectives on subtyping TTM and SPD. For example, Grant et al. [71] presented three separate subtypes for TTM (i.e., “sensory sensitive pullers”, “low awareness pullers” and “impulsive/perfectionist pullers”) and two separate subtypes for SPD (i.e., “emotional/reward pickers” and “functional pickers”) on the basis of their scores on MIST-A, MIDAS, MGH-HPS, and SPS-R. The relationships of these subtypes with EMSs have not yet been explored.

## Conclusion

The lack of between-group differences in EMS levels between individuals with TTM, SPD, and OCD corroborates the transdiagnostic function of EMSs. Several EMSs were significantly positively associated with self-reported and clinician-rated symptoms and picking/pulling styles of TTM and SPD, with some indications of diagnosis-specific association patterns. These results suggest that EMSs are common among individuals with TTM and SPD and that the assessment of EMSs may be of relevance and help to clinicians’ understanding of TTM and SPD patients’ presenting problems. However, as only a limited number of studies on the role of EMSs in TTM and SPD exist thus far, further research should continue to investigate their associations with TTM and SPD severity and subtypes in large clinical samples of TTM and SPD patients. Longitudinal treatment studies would be of particular interest to increase our knowledge of their temporal associations, as well as EMSs’ potential relationships with TTM and SPD treatment outcomes. If future research demonstrates that EMSs are predictors of treatment outcomes for TTM and SPD, it would indicate that greater emphasis should be placed on addressing personality problems in therapy to enhance treatment effectiveness.

## Data Availability

Due to restrictions imposed by the Regional Medical Ethics Committee to protect patient confidentiality, the data are available only upon reasonable request. Requests should be directed to the Privacy and Data Protection Officer at Oslo University Hospital, Oslo, Norway, personvern@ous-hf.no.

## Footnotes

1 The total number exceeds 59 because five patients had two comorbid diagnoses.

## References

[1] American Psychiatric Association. Diagnostic and statistical manual of mental disorders. 5th ed. American Psychiatric Association; 2013.

[2] World Health Organization. (2019). *International statistical classification of diseases and related health problems* (11th ed.). https://icd.who.int/10.2471/BLT.25.294650 PMC13531092 42683092

[3] Grant JE, Dougherty DD, Chamberlain SR. Prevalence, gender correlates, and co-morbidity of trichotillomania. Psychiatry Res. 2020;288:112948–112948. 10.1016/j.psychres.2020.112948. 32334275 10.1016/j.psychres.2020.112948 PMC7212053

[4] Grant JE, Chamberlain SR. Prevalence of skin picking (excoriation) disorder. J Psychiatr Res. 2020;130:57–60. 10.1016/j.jpsychires.2020.06.033. 32781374 10.1016/j.jpsychires.2020.06.033 PMC7115927

[5] Snorrason I, Belleau EL, Woods DW. How related are hair pulling disorder (trichotillomania) and skin picking disorder? A review of evidence for comorbidity, similarities and shared etiology. Clin Psychol Rev. 2012;32(7):618–29. 10.1016/j.cpr.2012.05.008. 22917741 10.1016/j.cpr.2012.05.008

[6] Odlaug BL, Grant JE. Trichotillomania and pathologic skin picking: clinical comparison with an examination of comorbidity. Annals Clin Psychiatry: Official J Am Acad Clin Psychiatrists. 2008;20(2):57–63. 10.1080/10401230802017027. 10.1080/10401230802017027 18568576

[7] Lin A, Farhat LC, Flores JM, Levine JLS, Fernandez TV, Bloch MH, Olfson E. Characteristics of trichotillomania and excoriation disorder across the lifespan. Psychiatry Res. 2023;322:115120. 10.1016/j.psychres.2023.115120. 36842397 10.1016/j.psychres.2023.115120 PMC10023474

[8] Flessner CA, Woods DW, Franklin ME, Cashin SE, Keuthen NJ, Trichotillomania Learning Center-Scientific Advisory Board. The Milwaukee Inventory for Subtypes of Trichotillomania-Adult Version (MIST-A).: Development of an Instrument for the Assessment of Focused and Automatic Hair Pulling. J Psychopathol Behav Assess. 2008;30(1):20–30. 10.1007/s10862-007-9073-x

[9] Grant JE, Chamberlain SR. Automatic and focused hair pulling in trichotillomania: valid and useful subtypes? Psychiatry Res. 2021b;306:114269. 10.1016/j.psychres.2021.114269. 34758405 10.1016/j.psychres.2021.114269 PMC7612152

[10] Walther MR, Flessner CA, Conelea CA, Woods DW. The Milwaukee inventory for the dimensions of adult skin picking (MIDAS): initial development and psychometric properties. J Behav Ther Exp Psychiatry. 2009;40(1):127–35. 10.1016/j.jbtep.2008.07.002. 18725154 10.1016/j.jbtep.2008.07.002

[11] Christenson GA, Mackenzie TB. Trichotillomania. In: Hersen M, Ammerman RT, editors. Handbook of prescriptive treatment for adults. New York, NY: Plenum; 1994. pp. 217–35.

[12] Gallinat C, Moessner M, Wilhelm M, Keuthen N, Bauer S. Patterns of skin picking in skin picking disorder: ecological momentary assessment study. Interact J Med Res. 2024;13:e53831. 10.2196/53831. 39024568 10.2196/53831 PMC11294777

[13] Flessner CA, Conelea CA, Woods DW, Franklin ME, Keuthen NJ, Cashin SE. Styles of pulling in trichotillomania: exploring differences in symptom severity, phenomenology, and functional impact. Behav Res Ther. 2008;46(3):345–57. 10.1016/j.brat.2007.12.009. 18249363 10.1016/j.brat.2007.12.009

[14] Schienle A, Zorjan S, Übel S, Wabnegger A. Prediction of automatic and focused skin picking based on trait disgust and emotion dysregulation. J Obsessive-Compulsive Relat Disorders. 2018;16:1–5. 10.1016/j.jocrd.2017.10.006.

[15] Barber KE, Cram IF, Smith EC, Capel LK, Snorrason I, Woods DW. Anxiety and body-focused repetitive behaviors: A systematic review and meta-analysis of comorbidity rates and symptom associations. J Psychiatr Res. 2025;181:80–90. 10.1016/j.jpsychires.2024.11.062. 39603165 10.1016/j.jpsychires.2024.11.062

[16] Grant JE, Chamberlain SR. Personality traits and their clinical associations in trichotillomania and skin picking disorder. BMC Psychiatry. 2021;21(1):203. 10.1186/s12888-021-03209-y. 33882867 10.1186/s12888-021-03209-y PMC8059235

[17] Keuthen NJ, Tung ES, Altenburger EM, Blais MA, Pauls DL, Flessner CA. Trichotillomania and personality traits from the five-factor model. Brazilian J Psychiatry. 2015;37(4):317–24. 10.1590/1516-4446-2015-1657. 10.1590/1516-4446-2015-1657 26375807

[18] Haaland AT, Vogel PA, Launes G, Haaland VØ, Hansen B, Solem S, Himle JA. The role of early maladaptive schemas in predicting exposure and response prevention outcome for obsessive-compulsive disorder. Behav Res Ther. 2011;49(11):781–8. 10.1016/j.brat.2011.08.007. 21920500 10.1016/j.brat.2011.08.007

[19] Young JE, Klosko JS, Weishaar ME. Schema therapy: A practitioners guide. New York: Guilford; 2003.

[20] Bishop A, Younan R, Low J, Pilkington PD. Early maladaptive schemas and depression in adulthood: A systematic review and meta-analysis. Clin Psychol Psychother. 2022;29(1):111–30. 10.1002/cpp.2630. 34131990 10.1002/cpp.2630

[21] Tariq A, Quayle E, Lawrie SM, Reid C, Chan SWY. Relationship between early maladaptive schemas and anxiety in adolescence and young adulthood: A systematic review and meta-analysis. J Affect Disord. 2021;295:1462–73. 10.1016/j.jad.2021.09.031. 34563389 10.1016/j.jad.2021.09.031

[22] Bortolon C, Capdevielle D, Boulenger J-P, Gely-Nargeot M-C, Raffard S. Early maladaptive schemas predict positive symptomatology in schizophrenia: A cross-sectional study. Psychiatry Res. 2013;209(3):361–6. 10.1016/j.psychres.2013.03.018. 23623454 10.1016/j.psychres.2013.03.018

[23] Dostal AL, Pilkington PD. Early maladaptive schemas and obsessive-compulsive disorder: A systematic review and meta-analysis. J Affect Disord. 2023;336:42–51. 10.1016/j.jad.2023.05.053. 37217101 10.1016/j.jad.2023.05.053

[24] Stein DJ, Simeon D, Cohen LJ, Hollander E. Trichotillomania and obsessive-compulsive disorder. The J Clin Psychiatry 56 Suppl. 1995;4:28–35. 10.1176/ajp.150.7.1131-a. 10.1176/ajp.150.7.1131-a 7713862

[25] Tükel R, Keser V, Karali NT, Olgun TO, Calikuşu C. Comparison of clinical characteristics in trichotillomania and obsessive-compulsive disorder. J Anxiety Disord. 2001;15(5):433–41. 10.1016/s0887-6185(01)00074-3. 11583075 10.1016/s0887-6185(01)00074-3

[26] Thimm JC, Chang M. Early maladaptive schemas and mental disorders in adulthood: A systematic review and meta-analysis. Int J Cogn Therapy. 2022;15(4):371–413. 10.1007/s41811-022-00149-7.

[27] Bär A, Bär HE, Rijkeboer MM, Lobbestael J. Early maladaptive schemas and Schema modes in clinical disorders: A systematic review. Psychol Psychother. 2023;96(3):716–47. 10.1111/papt.12465. 37026578 10.1111/papt.12465

[28] Sunde T, Hummelen B, Himle JA, Walseth LT, Vogel PA, Launes G, Haaland V, Haaland Å, T. Early maladaptive schemas impact on long-term outcome in patients treated with group behavioral therapy for obsessive-compulsive disorder. BMC Psychiatry. 2019;19(1):318. 10.1186/s12888-019-2285-2. 31655556 10.1186/s12888-019-2285-2 PMC6815412

[29] Lochner C, Seedat S, du Toit PL, Nel DG, Niehaus DJ, Sandler R, Stein DJ. Obsessive-compulsive disorder and trichotillomania: a phenomenological comparison. BMC Psychiatry. 2005;5:2. 10.1186/1471-244x-5-2. 15649315 10.1186/1471-244X-5-2 PMC546013

[30] Pozza A, Albert U, Dèttore D. Early maladaptive schemas as common and specific predictors of skin picking subtypes. BMC Psychol. 2020;8(1):27. 10.1186/s40359-020-0392-y. 32188511 10.1186/s40359-020-0392-y PMC7081682

[31] Young JE, Brown G. Young Schema Questionnaire-Short form. New York: Schema Therapy Institute; 1998. http://www.schematherapy.com.

[32] Arabatzoudis T, Rehm IC, Nedeljkovic M, Moulding R. Emotion regulation in individuals with and without trichotillomania. J Obsessive-Compulsive Relat Disorders. 2017;12:87–94. 10.1016/j.jocrd.2017.01.003.

[33] Keeler SD. (2018). Attachment Style and Characteristics of Automatic and Focused Skin Picking. Doctoral dissertation, The Chicago School of Professional Psychology.

[34] Goodman WK, Price LH, Rasmussen SA, Mazure C, Fleischmann RL, Hill CL, Heninger GR, Charney DS. The Yale-Brown obsessive compulsive scale: I. Development, use, and reliability. Arch Gen Psychiatry. 1989;46(11):1006–11. 10.1001/archpsyc.1989.01810110048007. 2684084 10.1001/archpsyc.1989.01810110048007

[35] Haaland ÅT, Eskeland SO, Moen EM, Vogel PA, Haseth S, Mellingen K, Himle JA, Woods DW, Hummelen B. ACT-enhanced behavior therapy in group format for trichotillomania: an effectiveness study. J Obsessive-Compulsive Relat Disorders. 2017;12:109–16. 10.1016/j.jocrd.2017.01.005.

[36] World Medical Association. World medical association declaration of helsinki: ethical principles for medical research involving human subjects. JAMA. 2013;310(20):2191–4. 10.1001/jama.2013.281053. 24141714 10.1001/jama.2013.281053

[37] Brown T, Di Nardo P, Barlow D. Anxiety disorders interview schedule for DSM-IV (ADIS-IV). Diagnostic assessment instrument. Oxford University Press; 1994.

[38] Sheehan DV, Lecrubier Y, Sheehan KH, Amorim P, Janavs J, Weiller E, Hergueta T, Baker R, Dunbar GC. The Mini-International neuropsychiatric interview (M.I.N.I.): the development and validation of a structured diagnostic psychiatric interview for DSM-IV and ICD-10. J Clin Psychiatry. 1998;59(20):22–57. 9881538

[39] Rothbaum BO, Ninan PT. The assessment of trichotillomania. Behav Res Ther. 1994;32(6):651–62. 10.1016/0005-7967(94)90022-1. 8085996 10.1016/0005-7967(94)90022-1

[40] Grant JE, Stein DJ, Woods D, Keuthen N. Trichotillomania, skin picking, and other Body-focused repetitive behaviors. Arlington, VA, USA: American Psychiatric Publishing, Inc; 2012.

[41] First MB, Spitzer RL, Gibbon M, Williams JBW. (1995). Structured clinical interview for DSM-IV axis I disorders patient edition (SCID I/P, version 2.0). NY: Biometrics Research Department.

[42] Young JE, Brown G. Young Schema questionnaire. New York: Schema Therapy Institute; 1990. http://www.schematherapy.com.

[43] Hoffart A, Sexton H, Hedley LM, Wang CE, Holthe H, Haugum JA, Nordahl HM, Hovland OJ, Holte A. The structure of maladaptive schemas: A confirmatory factor analysis and a psychometric evaluation of factor-Derived scales. Cogn Therapy Res. 2005;29(6):627–44. 10.1007/s10608-005-9630-0.

[44] Welburn K, Coristine M, Dagg P, Pontefract A, Jordan S. The Schema Questionnaire—Short form: factor analysis and relationship between schemas and symptoms. Cogn Therapy Res. 2002;26(4):519–30. 10.1023/A:1016231902020.

[45] Keuthen NJ, O’Sullivan RL, Ricciardi JN, Shera D, Savage CR, Borgmann AS, Jenike MA, Baer L. The Massachusetts general hospital (MGH) hairpulling scale: 1. development and factor analyses. Psychother Psychosom. 1995;64(3–4):141–5. 10.1159/000289003. 8657844 10.1159/000289003

[46] Barber KE, Woods DW, Bauer CC, Twohig MP, Saunders SM, Compton SN, Franklin ME. Psychometric properties of trichotillomania severity measures. Cogn Therapy Res. 2024;48(1):18–29. 10.1007/s10608-023-10406-4.

[47] O’Sullivan RL, Keuthen NJ, Hayday CF, Ricciardi JN, Buttolph ML, Jenike MA, Baer L. The Massachusetts general hospital (MGH) hairpulling scale: 2. reliability and validity. Psychother Psychosom. 1995;64(3–4):146–8. 10.1159/000289004. 8657845 10.1159/000289004

[48] Snorrason I, Ólafsson RP, Flessner CA, Keuthen NJ, Franklin ME, Woods DW. The skin picking Scale-Revised: factor structure and psychometric properties. J Obsessive-Compulsive Relat Disorders. 2012;1(2):133–7. 10.1016/j.jocrd.2012.03.001.

[49] Snorrason I, Keuthen NJ, Lee H-J, Beard C, Björgvinsson T. Skin picking Scale-Revised: diagnostic accuracy and optimal cut-off scores in university and psychiatric settings. J Obsessive-Compulsive Relat Disorders. 2022;34:100743. 10.1016/j.jocrd.2022.100743.

[50] Alexander JR, Houghton DC, Twohig MP, Franklin ME, Saunders SM, Neal-Barnett AM, Compton SN, Woods DW. Factor analysis of the Milwaukee inventory for subtypes of Trichotillomania-Adult version. J Obsessive-Compulsive Relat Disorders. 2016;11:31–8. 10.1016/j.jocrd.2016.08.001. 10.1016/j.jocrd.2016.08.001 PMC5033380 27668153

[51] Guy W. ECDEU assessment manual for psychopharmacology. Public Health Service: US Department of Health, Education, and Welfare; 1976.

[52] Bourredjem A, Pelissolo A, Rotge J-Y, Jaafari N, Machefaux S, Quentin S, Bui E, Bruno N, Pochon J-B, Polosan M, Baup N, Papetti F, Chéreau I, Arbus C, Mallet L., & du Moncel ST. A video clinical global impression scale (CGI) in obsessive compulsive disorder. Psychiatry Res. 2011;186(1):117–22. 10.1016/j.psychres.2010.06.021 20621362 10.1016/j.psychres.2010.06.021

[53] Kadouri A, Corruble E, Falissard B. The improved clinical global impression scale (iCGI): development and validation in depression. BMC Psychiatry. 2007;7:7. 10.1186/1471-244X-7-7. 17284321 10.1186/1471-244X-7-7 PMC1802073

[54] Zaider TI, Heimberg RG, Fresco D, Schneier F, Liebowitz M. Evaluation of the clinical global impression scale among individuals with social anxiety disorder. Psychol Med. 2003;33(4):611–22. 10.1017/S0033291703007414. 12785463 10.1017/s0033291703007414

[55] Diefenbach GJ, Tolin DF, Hannan S, Crocetto J, Worhunsky P. Trichotillomania: impact on psychosocial functioning and quality of life. Behav Res Ther. 2005;43(7):869–84. 10.1016/j.brat.2004.06.010. 15896284 10.1016/j.brat.2004.06.010

[56] Cervin M, Severity Benchmark OCD Consortium, Mataix-Cols D. Empirical severity benchmarks for obsessive-compulsive disorder across the lifespan. World Psychiatry: Official J World Psychiatric Association (WPA). 2022;21(2):315–6. 10.1002/wps.20984. 10.1002/wps.20984 PMC9077592 35524607

[57] Grabill K, Merlo L, Duke D, Harford KL, Keeley ML, Geffken GR, Storch EA. Assessment of obsessive-compulsive disorder: a review. J Anxiety Disord. 2008;22(1):1–17. 10.1016/j.janxdis.2007.01.012. 17367988 10.1016/j.janxdis.2007.01.012

[58] López-Pina JA, Sánchez-Meca J, López-López JA, Marín-Martínez F, Núñez-Núñez RM, Rosa-Alcázar AI, Gómez-Conesa A, Ferrer-Requena J. The Yale-Brown obsessive compulsive scale: A reliability generalization Meta-Analysis. Assessment. 2015;22(5):619–28. 10.1177/1073191114551954. 25268017 10.1177/1073191114551954

[59] Cohen J. A power primer. Psychol Bull. 1992;112(1):155–9. 10.1037/0033-2909.112.1.155. 19565683 10.1037//0033-2909.112.1.155

[60] Hawke LD, Provencher MD. Schema theory and schema therapy in mood and anxiety disorders: A review. J Cogn Psychother. 2011;25(4):257–76. 10.1891/0889-8391.25.4.257.

[61] Sundag J, Ascone L, de Matos Marques A, Moritz S, Lincoln TM. Elucidating the role of early maladaptive schemas for psychotic symptomatology. Psychiatry Res. 2016;238:53–9. 10.1016/j.psychres.2016.02.008. 27086211 10.1016/j.psychres.2016.02.008

[62] Weingarden H, Renshaw KD. Shame in the obsessive compulsive related disorders: A conceptual review. J Affect Disord. 2015;171:74–84. 10.1016/j.jad.2014.09.010. 25299438 10.1016/j.jad.2014.09.010 PMC4252512

[63] Kraemer HC, Kupfer DJ. Size of treatment effects and their importance to clinical research and practice. Biol Psychiatry. 2006;59(11):990–6. 10.1016/j.biopsych.2005.09.014. 16368078 10.1016/j.biopsych.2005.09.014

[64] Moir VK, Marais I, Lee CW. The effects of item placement in the young Schema questionnaire. J Appl Meas. 2017;18(4):370–82. 29252206

[65] Grant JE, Odlaug BL, Kim SW. A clinical comparison of pathologic skin picking and obsessive-compulsive disorder. Compr Psychiatr. 2010;51(4):347–52. 10.1016/j.comppsych.2009.10.006. 10.1016/j.comppsych.2009.10.006 20579505

[66] Capel LK, Petersen JM, Woods DW, Marcks BA, Twohig MP. Mental health providers’ knowledge of trichotillomania and skin picking disorder, and their treatment. Cogn Therapy Res. 2024;48:30–8. 10.1007/s10608-023-10381-w.

[67] Rehm IC, Nedeljkovic M, Moulding R, Thomas A. The beliefs in trichotillomania scale (BiTS): factor analyses and preliminary validation. Br J Clin Psychol. 2019;58(4):384–405. 10.1111/bjc.12219. 30968971 10.1111/bjc.12219

[68] Hutsebaut J, Bender DS. The clinical utility of the level of personality functioning scale: A treatment perspective. J Psychiatr Pract. 2024;30(6):411–20. 10.1097/PRA.0000000000000822. 39655967 10.1097/PRA.0000000000000822

[69] Giromini L, Young G, Sellbom M. Assessing negative response Bias using Self-Report measures: new articles, new issues. Psychol Injury Law. 2022;15(1):1–21. 10.1007/s12207-022-09444-2.

[70] Podsakoff PM, MacKenzie SB, Podsakoff NP. Sources of method bias in social science research and recommendations on how to control it. Ann Rev Psychol. 2012;63:539–69. 10.1146/annurev-psych-120710-100452. 21838546 10.1146/annurev-psych-120710-100452

[71] Grant JE, Peris TS, Ricketts EJ, Lochner C, Stein DJ, Stochl J, Chamberlain SR, Scharf JM, Dougherty DD, Woods DW, Piacentini J, Keuthen NJ. Identifying subtypes of trichotillomania (hair pulling disorder) and excoriation (skin picking) disorder using mixture modeling in a multicenter sample. J Psychiatr Res. 2021;137:603–12. 10.1016/j.jpsychires.2020.11.001. 33172654 10.1016/j.jpsychires.2020.11.001 PMC7610704

